# Tough Times Require Tough People: The Benefits of Grit for Reducing Employee Burnout

**DOI:** 10.3390/ijerph20116024

**Published:** 2023-06-01

**Authors:** Kari Kristinsson, Sigurdur Gudjonsson, Bryndis Kristjansdottir

**Affiliations:** School of Business, University of Iceland, 101 Reykjavik, Iceland

**Keywords:** burnout, grit, personality traits, emotional exhaustion, workload

## Abstract

Organizations are facing a serious challenge with employee burnout, which leads to a loss of productivity and employee morale. Despite its importance, there is still a knowledge gap in understanding one of the key features of employee burnout, namely, the personal characteristics of employees. This research aims to determine if grit can alleviate employee burnout in organizations. The study conducted a survey of employees in service companies, and results showed that employee grit was negatively associated with burnout. Moreover, the study revealed that grit does not equally affect all three dimensions of burnout, with emotional exhaustion and depersonalization being the most affected by employee grit. Increasing employee grit is therefore a promising strategy for companies that want to mitigate the risk of employee burnout.

## 1. Introduction

Poorly managed work environments can cause excessive stress for employees, leading to burnout [1,2]. Creating a healthy work environment benefits both employees and managers. Employees need to take responsibility for their own well-being by adopting healthy lifestyles, cultivating positive attitudes, and setting achievable goals, while managers need to foster positive environments within their organizations [3,4]. To promote employee well-being, managers can offer rewards, provide constructive feedback, and leverage employees’ strengths for the benefit of both the individual and the organization [5]. Although personality traits such as grit can help employees cope with stress and prevent burnout, it is crucial for employees to receive support, understanding, and professional backup from their management teams [6,7]. Despite a growing body of literature on burnout, there is a need for a better understanding of how personal characteristics of employees such as grit affects their propensity for burnout.

The objective of this study is to investigate the connection between grit and burnout by examining the sub-components of Maslach’s Burnout work list, namely emotional exhaustion, depersonalization, and personal achievement. The aim is to determine if grit has a protective effect against burnout at work and to deepen our understanding of this health problem. Specifically, this study seeks to explore whether participants who exhibit higher levels of grit are less likely to experience burnout at work. We define grit as a psychological construct that refers to an individual’s ability to persist and maintain effort towards achieving long-term goals, despite experiencing setbacks or obstacles. It involves the sustained application of effort and the ability to persevere through difficult circumstances. As prior literature reviews indicate, the concept of grit is highly related to other psychological variables, such as passion and a growth mindset [8]. Additionally, burnout is an important but understudied subject in service companies [9]. The main contribution of this research is therefore to investigate how the sub-components of grit are related to burnout in service companies, while controlling for related personal characteristics.

The remainder of this paper is structured as follows: we first present the theoretical background and relevant literature that underpins this research. Next, we describe the methodology of the study. We then present the results of our analysis and, finally, discuss the implications of our findings.

## 2. Grit and Burnout

Maslach and Leiter [10] identified several factors that must be balanced to promote workplace well-being, including a manageable workload, employee decision-making authority, and access to necessary resources. Additionally, appropriate rewards, managerial support, fairness, and control over one’s own progress are crucial for both employees and organizations. When these factors are in balance, employees are more likely to exhibit sanctity at work and less likely to experience burnout. However, despite these recommendations, workplace stress and strain have never been greater, with up to half of European employees reporting common stressors [11]. Moreover, younger employees entering the labour market are at a higher risk of depression, burnout, financial concerns, and unemployment, emphasizing the need for increased managerial support [12,13].

To prevent physical and mental problems, employees should receive appropriate training and career development, and there should be trust and encouragement between employees and managers. Additionally, managers must view workplace stress as an organizational issue rather than solely the employee’s fault, to better treat and prevent burnout [11]. Companies that promote well-being, health, and mental health can reduce stress and burnout while increasing productivity and safety, benefiting both employees and the organization [11,14].

The WHO defines burnout as a syndrome resulting from unmanaged chronic workplace stress, manifesting in emotional fatigue, suspicion, and decreased work performance [14]. Although not a disease, burnout is a syndrome that can lead to depression and negatively impact one’s health and job performance [15,16]. Younger individuals are more susceptible to burnout [10,17], which can be influenced by various factors, such as incompetence, insomnia, substance abuse, and family issues [18]. Burnout can be characterized by six key aspects: workload, management, reward, community, fairness, and value [10], all of which must be balanced to promote employee well-being and productivity [19]. Additionally, grit has been found to drive employees’ success in their field of interest [20] and should be considered in relation to burnout.

Research suggests that grit is a reliable predictor of one’s long-term goals [21,22] and is highly correlated with traits such as conscientiousness, willpower, self-discipline, and an even higher IQ [21,22,23]. Moreover, grit has been found to be a reliable predictor of individual performance [5].

Given the importance of grit in predicting success and performance, it is timely to investigate whether there is a relationship between grit and the three subdimensions of burnout (emotional exhaustion, depersonalization, and personal accomplishment). To the best of our knowledge, this is the first study to explore whether grit can serve as a protective factor against burnout in service-oriented companies.

In a study of medical graduates, Matero et al. [24] demonstrated that students with higher levels of grit performed better in challenging courses and were more likely to complete their studies in four years. Conversely, students with lower levels of grit were twice as likely to drop out of medical school [16]. Other research suggests that higher levels of grit can help prevent burnout among physician students [16] and salespeople [25]. Additionally, research in the health sector, as well as for students in general [26,27], has shown that grit can minimize burnout [22,28,29,30]. Despite the prevalence of burnout in service companies [9,31,32,33], there has been little research on the relationship between grit and burnout in this context.

## 3. Methods

### 3.1. Study Design, Participants and Pilot Study

A total of 14 service companies and institutions in Reykjavík, Iceland were contacted via email, of which seven agreed to participate in the study. The human resources managers of these companies were then sent the survey, which was subsequently forwarded to their employees. The survey was pre-tested with 10 parties prior to the study, and various changes were made. Participant responses were collected using Questionpro, and the research was conducted anonymously and was untraceable. Questionpro is an online survey software that allows users to create and distribute surveys, collect responses, and analyze data in real time. The survey took approximately 10–12 min to complete.

The convenience sample in the study comprised 598 employees from service companies located in Reykjavik. Of these, 144 participants responded, representing a reasonable response rate of 25%. The respondents included 43 men (38.1%), 68 women (60.2%), and 2 participants who did not disclose their gender identity (1.8%). Among the participants, 32.7% were managers or involved in human resources management, with 50% having completed a master’s or MBA program. A total of 25.9% had completed undergraduate degrees, 17.9% had completed high school or technical training, while 6 had only completed compulsory education and one had completed doctoral studies.

### 3.2. Measurments

The participants completed four questionnaires, including ones on grit and burnout using Maslach’s Burnout Inventory scale, as well as additional questions on their background.

#### 3.2.1. Grit

The Icelandic version of the grit scale [34] was utilized in this study, which was translated by Sigmundsson, Hagar and Hermundsdottir [20]. This scale is derived from the Duckworth persistence scale [23]. Participants were presented with eight statements and asked to indicate the extent to which they agreed or disagreed with each statement. The response options ranged from strongly disagree to strongly agree, with a five-point scale of strongly disagree, rather disagree, neither agree nor disagree, rather agree, or strongly agree. The scale comprises two sub-scales, each consisting of four questions. Each question has a maximum score of five, representing high grit, and a minimum score of one, representing no or little grit. To obtain an accurate measurement of participants’ grit, the questions that indicated lower grit were reversed. A higher average score on the scale indicates higher levels of grit. The Norwegian version, which the Icelandic translation is based on, has demonstrated evidence of internal consistency, test-retest consistency, predictive validity, and consensual validity. However, the questionnaire has not been localized for use in Iceland.

#### 3.2.2. Maslach Burnout Inventory

The MBI-HSS self-assessment scale [18] was utilized to measure job satisfaction, which is based on the Maslach Burnout Inventory (MBI) model and assesses burnout. The scale comprises 22 questions that are divided into three sub-scales, each measuring a dimension of burnout syndrome: emotional exhaustion, depersonalization, and personal accomplishment. Responses to the scale ranged from “never” (0 points) to “every day” (6 points) on a seven-point scale. Emotional exhaustion consists of nine statements that evaluate feelings or experiences at work, depersonalization includes five statements that measure dissatisfaction or impersonal reactions towards others, and personal achievement contains eight statements that assess an individual’s sense of competence and success at work. The higher the emotional exhaustion and depersonalization scores, the higher the probability of burnout, while the opposite is true for personal achievement. No total score was calculated, and instead, the subscales were used to evaluate burnout. Participants who scored low in emotional exhaustion and high in personal achievement were less likely to be experiencing burnout. The internal reliability of the subscales was good, and the test-retest reliability was also good. Additionally, the convergent validity of the subscales was considered good. Answering the scale takes approximately 8 to 10 min, and it is easy to administer. The MBI-HSS scale was translated into Icelandic by Rúnar Helgi Andrason and Sigurður Levy [35], but it has not been localized to the Icelandic population and thus cannot be used for clinical measurements. In Table 1, we provide interpretation criteria for the total score of the burnout inventory.

#### 3.2.3. Background Questions

A small number of background variables were included, such as gender, age when starting work, managerial roles, years of work experience, and education level. The analysis focused only on gender and age-related differences. The work experience variable was dichotomized into less than 10 years or more than 10 years. The 2016 salary survey conducted by VR was utilized, which reported an average of approximately 10 years of experience for directors, financial managers, store managers, and sales managers. Table 2 provides descriptive statistics for the main variables of interest in this research.

## 4. Results

### 4.1. Burnout

Out of all the participants, 66.7% reported not experiencing burnout, while 20% were unsure and 13.3% confirmed experiencing burnout at work. Men had a significantly lower likelihood of experiencing burnout compared to women.

The MBI-HSS self-assessment scale was used to assess job satisfaction, and the total score of each sub-scale was computed. Table 3 displays the distribution of participants across the sub-scales. In Table 2, we show the participant distribution on the Maslach scale.

Approximately 37.2% of participants showed low scores on emotional exhaustion, while 23.4% showed low scores on depersonalization, and 16% scored high on personal achievement. When considering the proportion of individuals who scored low on all three dimensions, only 4.5% experienced burnout. Additionally, 40% of participants aged 21–30 reported experiencing a high degree of emotional exhaustion, while only 20% of those aged 41–50 reported the same. 

### 4.2. Relationship between Burnout and Grit 

The study aimed to explore the correlation between burnout and grit using the Pearson correlation coefficient. Specifically, the correlation between the three subcategories of emotional exhaustion, depersonalization and personal achievement, and grit was investigated. Results showed a significant correlation between the three sub-components of Maslach’s job list and grit. More specifically, there was a significant negative correlation between emotional exhaustion (r = −0.316, *p* = 0.002) and depersonalization (r = −0.254, *p* = 0.011) and grit, and a significant positive correlation between personal achievement (r = 0.314, *p* = 0.002) and grit. 

Further analysis was conducted by gender, which revealed a significant negative correlation between grit and emotional exhaustion (r = −0.415, *p* = 0.013) and depersonalization (r = −0.352, *p* = 0.05) for men, but not personal achievement (r = −0.205, *p* = 0.218). On the other hand, women showed a significant positive correlation between personal achievement and grit (r = 0.359, *p* = 0.007), but not emotional exhaustion (r = −0.205, *p* = 0.142) or depersonalization (r = −0.142, *p* = 0.284).

Additionally, the relationship between emotional exhaustion and grit was examined by age and was divided into two variables: 21–40 years and 41 years and older. The results showed a significant negative correlation between emotional exhaustion and grit among participants aged 40 and younger (r = −0.482, *p* = 0.005), but not among participants aged 41 and older (r = −0.205, *p* = 0.122).

### 4.3. Maslach’s Three Dimensional Burnout Inventory (HBI-HSS) Scale: Emotional Exhaustion, Depersonalization and Personal Achievement

A linear regression analysis was conducted to analyze each dimension of the burnout inventory, using the following variables: grit, gender (male/female), education (with or without a university degree), managerial status (manager/non-manager), and work experience (less than 10 years/more than 10 years). Table 4 shows the dimensions of personal achievement, depersonalization and emotional exhaustion, as well as the grit variables and background variables.

The results of the linear regression analysis showed a significant association between emotional exhaustion and grit, with the model explaining 11.1% of the variability (R^2^ = 0.111 F (8 81) = 2.387, *p* < 0.052). As presented in Table 3, a one-point increase in grit was associated with a decrease in emotional exhaustion by 0.8. Additionally, the analysis revealed that being a manager and having worked for the company for more than 10 years were connected with emotional exhaustion.

Similarly, the results showed a significant association between depersonalization and grit, with the model explaining 9.7% of the variability (R^2^ = 0.097 F (8 89) = *p* < 0.027). As shown in Table 3, a one-point increase in grit was associated with a decrease in depersonalization by over 0.4. Furthermore, the analysis for depersonalization indicated a connection between being a manager and being under 40 years of age.

In summary, the findings suggest that grit has a strong relationship with emotional exhaustion and depersonalization.

## 5. Discussion and Further Research

The results suggest that individuals working in service companies in Iceland who possess high levels of grit are more likely to be satisfied with their work. This finding aligns with previous research in other industries [20,21,36]. Men exhibit greater passion and grit than women, as supported by prior studies [20,37].

Our study found that over half of the participants did not experience burnout, while 13% reported severe burnout, and 20% were uncertain if they had experienced burnout. These findings indicate a higher degree of burnout than previously reported by Gallup in Iceland. Moreover, men were nearly half as likely to experience burnout as women. Individuals 40 years of age and younger, who experience less grit and more emotional exhaustion, are likely to exhibit a negative relationship between grit and emotional exhaustion, according to the results. These results align with a Gallup study conducted in the United States [38]. The results further revealed that individuals with high scores on personal achievement are more satisfied with their work and perform well. Women are more likely to be satisfied with their personal achievements than men.

Individuals with grit are less likely to experience burnout. Those who exhibit emotional exhaustion, low depersonalization, and high personal achievement, indicating the absence of burnout at work, exhibit considerable grit. These results suggest that grit has a protective effect against burnout, which is consistent with prior research by Jumat et al. [16] and Duckworth [21]. Our linear regression analysis found a positive relationship between grit and the three dimensions of burnout, including emotional exhaustion, depersonalization, and personal achievement. The impact of grit was most significant on emotional exhaustion, followed by depersonalization and personal achievement. When examining all variables with the dimensions of the burnout inventory, 11.1% were explained by emotional exhaustion, 9.7% by depersonalization, and 23.6% by personal achievement. Future research should investigate these dimensions with additional variables in different industries, and, furthermore, given that remote work has become increasingly popular due to the COVID−19 pandemic, it is crucial to explore the impact of grit on remote workers’ burnout levels. Additionally, there is a need for further investigation into the relationship between grit and burnout in service companies and how these relate to dimensions of burnout, e.g., emotional exhaustion, depersonalization and personal achievement. Mixed-methods research could shed further light on the working conditions of service companies’ employees and their perceptions of grit and burnout by adding information about the human experience (qualitative data) to the study.

## 6. Conclusions

While our study only represents a preliminary step in comprehending the significance of employee grit in mitigating burnout, we believe that our findings have practical implications for management. As a result, we recommend that organizational management consider implementing employee grit training. Prior research suggests that this type of intervention could have considerable benefits [37,38] and may therefore represent a promising strategy for organizations seeking to reduce burnout among their workforces.

It is important to exercise caution when interpreting research findings. One limiting factor of our data is its subjective nature, as the burnout measure used reflects employees’ own subjective perception of their burnout levels. Ideally, a more objective measure would have been used, but this is difficult to achieve without physiological measures. However, prior research on burnout has used the same measures as we have done, which reinforces our result.

Another limitation of our research is that the data collected is self-reported and cross-sectional. While we found a distinct correlation between grit and employees’ burnout and its sub-dimensions, additional data gathering and analysis are required to validate these findings. An interesting research avenue could involve examining whether there is a causal relationship between grit and burnout. This could be achieved through a field experimental design, a method that has shown its value in other management research areas.

In conclusion, the study suggests that grit has a strong relationship with burnout, specifically emotional exhaustion and depersonalization. The findings highlight the importance of promoting grit among employees as a potential intervention to reduce burnout in the workplace. Furthermore, the study highlights the need for further research to investigate the factors that contribute to burnout among employees and to develop effective strategies to address this issue.

## Figures and Tables

**Table 1:** Interpretation criteria for the total score of the burnout inventory.

	Low	Middle	High
Emotional exhaustion	<18	19–26	>27
Depersonalization	<5	6–9	>10
Personal achievement	<40	39–34	>33

**Table 2:** Descriptive statistics for the main variables of interest.

	Age	Education	Gender	Grit	Manager
Mean	1.69	1.77	1.61	28.01	1.67
Median	2.00	2.00	2.00	28.00	2.00
Standard deviation	0.46	0.42	0.49	4.28	0.47

**Table 3:** Participant distribution on the Maslach scale.

	Low	Medium	High
	N (%)	N (%)	N (%)
Emotional Exhaustion	29 (30.9)	30 (31.9)	35 (37.2)
Depersonalization	48 (44.9)	34 (31.8)	25 (23.4)
Personal Achievements	14 (14)	70 (70)	16 (16)

**Table 4:** The dimensions of personal achievement, depersonalization and emotional exhaustion, as well as the grit variables and background variables.

	Emotional Exhaustion			Depersonalization			Personal Achievement		
	*B*	*SE*	β	*B*	*SE*	β	*B*	*SE*	β
*(Constant)*	430.3	120.9		280.7	60.9		150.8	10.4	
*Gr. Mindset*	0.17	0.19	0.10	0.005	0.10	0.006	0.301	0.155	0.20 *
*Passion*	0.022	0.21	0.014	0.094	0.11	0.10	0.417	0.165	0.281 **
*Grit*	−0.80	0.27	−0.33 ***	−0.42	0.14	−0.32 **	0.382	0.215	0.176 *
*Age*	−10.5	20.39	0.07	−20.3	10.3	−0.19 *	−0.39	10.93	0.02
*Gender*	0.90	20.1	0.05	−10.4	10.13	−0.12	−20.2	10.69	−0.12
*Education*	−0.52	20.54	−0.021	−0.25	10.4	−0.02	0.89	20.05	0.040
*Manager*	−40.7	20.28	−0.22 *	−20.8	10.2	−0.23 **	−30.9	10.84	−0.20 **
*Work Exp.*	40.2	20.4	0.20 *	0.35	10.3	0.03	−10.4	10.9	−0.071
*F*		20.4			20.3			40.68	
*Adjusted R^2*		0.0111			0.97			0.236	

*p* < 0.1 * *p* < 0.05 ** *p* < 0.01 ***.

## Data Availability

Not applicable.

## References

[1] Rasool S.F., Wang M., Tang M., Saeed A., Iqbal J. (2021). How Toxic Workplace Environment Effects the Employee Engagement: The Mediating Role of Organizational Support and Employee Wellbeing. Int. J. Environ. Res. Public Health.

[2] Rasool S.F., Wang M., Zhang Y., Samma M. (2020). Sustainable Work Performance: The Roles of Workplace Violence and Occupational Stress. Int. J. Environ. Res. Public Health.

[3] Kalinienė G., Lukšienė D., Ustinavičienė R., Škėmienė L., Januškevičius V. (2021). The burnout syndrome among women working in the retail network in associations with psychosocial work environment factors. Int. J. Environ. Res. Public Health.

[4] Lakiša S., Matisāne L., Gobiņa I., Vanadziņš I., Akūlova L., Eglīte M., Paegle L. (2021). Impact of workplace conflicts on self-reported medically certified sickness absence in Latvia. Int. J. Environ. Res. Public Health.

[5] Calo M., Peiris C., Chipchase L., Blackstock F., Judd B. (2019). Grit, resilience and mindset in health students. Clin. Teach..

[6] Arakelian E., Hellman T., Svartengren M. (2020). Experiences of the Initial Phase Implementation of the STAMINA-Model in Perioperative Context Addressing Environmental Issues Systematically—A Qualitative Study. Int. J. Environ. Res. Public Health.

[7] Toderi S., Balducci C. (2018). Stress-Preventive Management Competencies, Psychosocial Work Environments, and Affective Well-Being: A Multilevel, Multisource Investigation. Int. J. Environ. Res. Public Health.

[8] Schimschal S.E., Visentin D., Kornhaber R., Cleary M. (2021). Grit: A concept analysis. Issues Ment. Health Nurs..

[9] Humborstad S.I., Humborstad B., Whitfield R. (2007). Burnout and service employees’ willingness to deliver quality service. J. Hum. Resour. Hosp. Tour..

[10] Maslach C., Leiter M.P. (2016). Understanding the burnout experience: Recent research and its implications for psychiatry. World Psychiatry.

[11] Nielsen K., Jørgensen M.B., Milczarek M., Munar L. (2018). Healthy Workers, Thriving Companies—A Practical Guide to Wellbeing at Work. Tackling Psychosocial Risks and Musculoskeletal Disorders in Small Businesses [Brochure].

[12] Hampson E., Jacob A. (2020). Mental Health and Employers: Refreshing the Case for Investment.

[13] Schwartz J., Hatfield S., Jones R., Anderson S. (2019). What is the Future of Work? Redefining Work, Workforces and Workplace.

[14] World Health Organization (2019). Burn-Out an Occupational Phenomenon International Classification of Disease. https://www.who.int/news/item/28-05-2019-burn-out-an-occupational-phenomenon-international-classification-of-diseases.

[15] Baugh J.J., Takayesu J.K., White B.A., Raja A.S. (2020). Beyond the Maslach burnout inventory: Addressing emergency medicine burnout with Maslach’s full theory. Physician Wellness.

[16] Jumat M.R., Chow P.K., Allen J.C., Lai S.H., Hwang N.C., Iqbla J., Mok S.U.M., Rapisarda A., Velkey J.M., Engle D.L. (2020). Grit protects medical students from burnout: A longitudinal study. BMC Med. Educ..

[17] Pendell R. (2018). Millennials Are Burning Out.

[18] Maslach C., Jackson S.E., Leiter M.P. (1997). Maslach Burnout Inventory.

[19] De Vroome E. (2006). Prevalence of Sickness Absence and Presenteeism. Eurofound. https://www.eurofound.europa.eu/publications/article/2006/prevalence-of-sickness-absence-and-presenteeism.

[20] Sigmundsson H., Haga M., Hermundsdottir F. (2020). Passion, grit and mindset in young adults: Exploring the relationship and gender differences. New Ideas Psychol..

[21] Duckworth A.L. (2016). Grit: The Power of Passion and Perseverance.

[22] Dam A., Perera T., Jones M., Haughy M., Gaeta T. (2018). The Relationship Between Grit, Burnout and Well Being in Emergency Medicine Residents. AEM Educ. Train..

[23] Duckworth A.L., Peterson C., Matthews M.D., Kelly D.R. (2007). Grit: Perseverance and passion for long-time goals. J. Personal. Soc. Psychol..

[24] Matero L.R., Martinez S., MacLean L., Yaremchuk K., Ko A.B. (2018). Grit: A Predictor of Medical Student Performance. Educ. Health.

[25] Dugan R., Hochstein B., Rouziou M., Britton B. (2019). Gritting their teeth to close the sale: The positive effect of salesperson grit on job satisfaction and performance. J. Pers. Sell. Sales Manag..

[26] Tang X., Upadyaya K., Salmela-Aro K. (2021). School burnout and psychosocial problems among adolescents: Grit as a resilience factor. J. Adolesc..

[27] Teuber Z., Nussbeck F.W., Wild E. (2020). The Bright Side of Grit in Burnout-Prevention: Exploring Grit in the Context of Demands-Resources Model among Chinese High School Students. Child Psychiatry Hum. Dev..

[28] Brateanu A., Switzer B., Scott S.C., Ramsey J., Thomascik J., Nowacki A.S. (2020). Higher grit scores associated with less burnout in a cohort of internal medicine residents. Am. J. Med. Sci..

[29] Shakir H.J., Cappuzzo J.M., Shallwani H., Kwasnicki A., Bullis C., Wang J., Hess R.M., Levy E.I. (2020). Relationship of Grit and Resilience to Burnout Among U.S. Neurosurgery Residents. World Neurosurg..

[30] Halliday L., Walker A., Vig S., Hines J., Brecknell J. (2017). Grit and burnout in UK doctors: A cross-sectional study across specialties and stages of training. Postgrad. Med. J..

[31] Hsieh Y., Hsieh A. (2003). Does job standardization increase job burnout?. Int. J. Manpow..

[32] Suzabar D.F., Soelton M., Imaningsih E.S., Hutagalung I., Suherman A.D. (2020). Conceptualizing the role of self-esteem in the burnout process. Manag. Sci. Lett..

[33] Yagil D. (2006). The relationship of service provider power motivation, empowerment and burnout to customer satisfaction. Int. J. Serv. Ind. Manag..

[34] Duckworth A.L., Quinn P.D. (2009). Development and Validation of the Short Grit Scale (Grit-S). J. Personal. Assess..

[35] Haarde H.L. (2009). Viðhorf Lögreglumanna Til Þolenda Afbrota og Tengsl Þeirra við Starfsþrot. Óbirt BS—Ritgerð. Sótt af. https://skemman.is/bitstream/1946/2787/2/Helga%20Lara%20Haarde%20ritgerd_fixed.pdf.

[36] Muenks K., Canning E.A., Lacosse J., Dorainne J.G., Zirkel S., Garica J.A., Murphy M.C. (2020). Does my professor think my ability can change? Students’ perceptions of their STEM professors’ mindset beliefs predict their psychological vulnerability, engagement, and performance in class. J. Exp. Psychol. Gen..

[37] Sigmundsson H. (2021). Passion, grit and mindset in the ages 14 to 77: Exploiting relationship and gender differences. New Ideas Psychol..

[38] Harter J. (2020). 4 Factors Driving Records-High Employee Engagement in U.S. https://www.gallup.com/workplace/284180/factors-driving-record-high-employee-engagement.aspx.

